# Changes in Corneal Biomechanical Properties after Long-Term Topical Prostaglandin Therapy

**DOI:** 10.1371/journal.pone.0155527

**Published:** 2016-05-17

**Authors:** Na Wu, Yuhong Chen, Xiaobo Yu, Mengwei Li, Wen Wen, Xinghuai Sun

**Affiliations:** 1 Department of Ophthalmology and Visual Science, Eye and ENT Hospital of Fudan University, Shanghai, China; 2 Key Laboratory of Myopia, Ministry of Health (Fudan University), Shanghai, China; 3 Shanghai Key Laboratory of Visual Impairment and Restoration (Fudan University), Shanghai, China; 4 State Key Laboratory of Medical Neurobiology, Institutes of Brain Science, Fudan University, Shanghai, China; University of Oklahoma Health Sciences Center, UNITED STATES

## Abstract

**Objective:**

To compare corneal biomechanical properties, measured by a newly developed tonometer (Corneal Visualization Scheimpflug Technology, Corvis ST), in untreated primary open angle glaucoma (POAG) patients, POAG patients with long-term topical prostaglandin analog (PGA) therapy and in normal controls. Further is to investigate the potential effects of PGA on corneal biomechanics.

**Methods:**

In this case-control study, 35 consecutive medication naïve eyes with POAG, 34 POAG eyes with at least 2 years treatment by PGA and 19 normal eyes were included. Intraocular pressure (IOP), central corneal thickness (CCT) and corneal biomechanical parameters, including deformation amplitude (DA), applanation time (AT1 and AT2), applanation length (AL1 and AL2), applanation velocity (AV1 and AV2), and peak distance and radius were measured using Corvis ST. Axial length and corneal curvature were measured with partial coherence interferometry (IOLMaster, Zeiss, Germany). General linear model analysis was performed to investigate the corneal biomechanical property changes among the normal controls, newly diagnosed POAG patients and POAG patients with long-term PGA treatment, and among the subgroups of different types of PGA treatment, including bimatoprost, latanoprost and travoprost. Furthermore, pairwise comparisons using Bonferroni correction for least squares means were employed.

**Results:**

AT1 (p<0.0001), AV1 (p<0.0001), AT2 (p = 0.0001), AV2 (p<0.0001) and DA (p = 0.0004) in newly diagnosed glaucoma patients were significantly different from those in normal subjects and in patients underwent at least 2 years topical PGA therapy after adjusting for age and gender. After adjusting for age, gender, IOP, CCT, axial length and corneal curvature, a significant difference was detected for DA between glaucoma patients without PGA treatment and patients with long-term PGA therapy (p = 0.0387). Furthermore, there were no statistical significant differences in all of the corneal biomechanical parameters among the 3 types of PGA therapy subgroups, namely bimatoprost, latanoprost and travoprost.

**Conclusions:**

Significant changes in corneal deformation parameters were found among untreated POAG patients, POAG patients with long-term topical PGA therapy and normal controls. Long-term topical PGA treatment might have a direct effect on corneal biomechanical properties in addition to the indirect effect owing to the PGA-induced IOP reduction and CCT decrease on corneal dynamic properties.

## Introduction

Glaucoma is a leading cause of visual impairment and blindness worldwide. It has been universally acknowledged that intraocular pressure (IOP) is the most important risk factor for the occurrence and progression of glaucoma [1]. In most cases, topical medical therapy is the initial treatment for glaucoma [2]. The prostaglandin analogs (PGA) are highly effective first-line anti-glaucoma agents. Besides the ocular hypotensive effect, PGA can decrease the central corneal thickness (CCT) after long-term topical usage [3, 4]. CCT reduction may result from the degradation of collagen owing to activation of prostaglandin E receptors in the corneal stroma [3].

Alterations of corneal biomechanical properties have an effect on the progression of some ocular diseases including glaucoma [5]. IOP and CCT are both related to the biomechanical properties of the cornea. Higher IOP leads to stiffer corneal biomechanical behavior [5]. Corneal hysteresis (CH), measured by Ocular Response Analyzer (ORA), reflects corneal viscoelastic properties [6]. It has been reported that reductions of IOP accompanies with increases of CH [6]. CCT also plays an important role in corneal elastic properties. Studies have proposed that CCT has a strong correlation with CH in normal and glaucoma eyes [7–9].

The Corneal Visualization Scheimpflug Technology (Corvis ST) is a novel noncontact device that allows investigation of the biomechanical properties of human cornea *in vivo* [10]. The precision of Corvis ST was excellent in measuring IOP, CCT and time related corneal biomechanical parameters, and was good for velocity parameters, while moderate to fair for length parameters [11]. It has been reported that topical PGA treatment increases the CH measured by ORA [6, 9, 12], and this increase is not related to the reduction magnitude of the IOP induced by the therapy [6]. However, investigations on changes in corneal biomechanical parameters measured by Corvis ST after long-term therapy of topical PGA have not yet been reported.

The purpose of this study was to compare the corneal dynamic properties between groups of normal subjects, newly diagnosed open angle glaucoma patients and patients with long-term therapy of topical PGA, and to evaluate the potential effects of PGA on corneal biomechanical properties without involvement of its IOP and CCT reduction effects.

## Materials and Methods

This study was approved by the Medical Ethics Committee of the Shanghai Eye, Ear, Nose and Throat Hospital, and was carried out in accordance with the Declaration of Helsinki. Written confirmed consent was obtained from each participant before the examination.

This was an observational case control study. 19 normal controls, 35 newly diagnosed primary open angle glaucoma (POAG) patients without any previous medical, laser or surgical treatment and 34 POAG patients who had used only 1 type of topical PGA medications for at least 2 years, were consecutively enrolled from October 2014 to April 2015 at Shanghai Eye & ENT Hospital. Normal subjects did not have a previous history of glaucoma. Exclusion criteria included any corneal diseases, any previous history of ocular laser or surgical treatment, spherical refraction error less than -6.0 diopters (D) or more than +3.0D, and astigmatic refraction error greater than 2.0D.

Only one eye of each subject was allowed to be included in this study. The left eye was included when both eyes met the inclusion criteria. A full ophthalmologic examination was performed on all participants, including visual acuity, slit-lamp biomicroscopy, fundus evaluation with a 90D lens, and gonioscopy. Axial length and corneal curvature were measured on all participants with partial coherence interferometry (IOLMaster, Zeiss, Germany). IOP and CCT assessments were obtained using Corvis ST (OCULUS GmbH, Wetzlar, Germany).

Corvis ST was recently introduced as a novel noncontact tonometer. It takes images with a high-speed Scheimpflug camera to record the dynamic reaction of the cornea during an air puff application. After each measurement, corneal biomechanical parameters are produced as follows: applanation time, applanation length and velocity of the first (from start until reaching first applanation) and second (from start until reaching second applanation) corneal applanations (AT1, AL1, AV1 and AT2, AL2, AV2, respectively) and time, peak distance (PD), radius, deformation amplitude (DA) of the highest concavity. IOP and CCT were simultaneously recorded during each measurement process. In addition, age and gender of participants were collected before measurements.

Statistical analysis was performed using SPSS for Windows, version 17.0 (SPSS Inc., Chicago, IL). Data were shown as mean ± standard deviation (SD). Analysis of variance (ANOVA) with Bonferroni-adjusted post hoc comparisons was used to investigate the differences of biographical and clinical factors if the P value is more than 0.05 in the test of homogeneity of variances. If not, Tamhane’s T2-adjusted post hoc test was applied. The differences of corneal biomechanical properties were analyzed with the general linear model. Furthermore, pairwise comparisons using Bonferroni correction for least squares means were employed. It was considered to be statistically significant when the P values were less than 0.05.

## Results

A total of 19 normal eyes (19 controls, 9 males), 35 newly diagnosed POAG eyes (35 patients, 21 males) without PGA therapy and 34 POAG eyes (34 patients, 21 males) with only 1 type of long-term PGA anti-glaucoma treatment were included. Detailed information about the application years and type of PGA medications used in glaucoma patients is shown in Table 1. Travoprost was the main type of PGA medication used in glaucoma patients (47%, 16/34), followed by latanoprost (26.5%, 9/34) and bimotoprost (26.5%, 9/34). The average years of PGA treatment were 3.34 ± 1.79 (range: 2–9 years), and no statistical significant difference was found in application years among the 3 types of PGA medications (p = 0.964).

**Table 1:** Type of topical PGA medications used in patients with POAG.

	Number	Year, mean± SD	Range (years)
Bimatoprost	9	3.39±1.67	2–7
Latanoprost	9	3.44±1.67	2–7
Travoprost	16	3.25±2.01	2–9
Total	34	3.34±1.79	2–9
***P* value**		0.964	

Values are described as mean ± standard deviation. *P* value was calculated using one-way analysis of variance for usage years of topical PGA treatment.

Biographical and clinical details of all participants are summarized in Table 2. Mean age and gender distribution were similar among the normal controls, newly diagnosed patients without PGA therapy and eyes with long-term PGA treatment (p = 0.166 and 0.576, respectively). There were no statistical significant differences in corneal curvature (p = 0.989) and axial length (p = 0.053) among the 3 groups. Even though a slight decrease in average CCT was detected in patients with long-term treatment of PGA compared to the other 2 groups, no statistical significant difference was found among these groups (p = 0.061). The mean value of IOP (18.38±4.58, p<0.0001) in newly diagnosed glaucoma patients was significantly higher than that in the other 2 groups, while there was no statistical significant difference between the normal controls and patients with long-term treatment of only 1 type of PGA medication (14.70±2.85 and 12.91±2.75, respectively. Table 2). The same statistical results were obtained on the cornea compensated IOP (IOPcc) values (Table 2).

**Table 2:** Clinical factors in the normal eyes, POAG eyes without PGA treatment and eyes with long-term treatment of PGA.

Clinical factors	Overall sample (N = 88)	Normal subjects (N = 19)	Eyes without PGA treatment (N = 35)	Eyes with PGA treatment (N = 34)	*P* value
Age, years	49.40±14.41	44.95±14.63	48.69±15.35	52.62±12.86	0.166
Male, n (%)	51 (58%)	9 (47%)	21 (60%)	21 (62%)	0.576
IOP, mmHg	15.47±4.34	14.70±2.85	18.38±4.58	12.91±2.75	**<0.0001**
IOPcc, mmHg	14.71±4.09	13.32±3.17	17.17±4.41	12.96±2.80	**<0.0001**
CCT, μm	555.73±36.16	564.45±36.79	562.07±31.98	544.34±37.89	0.061
Axial Length, mm	24.56±1.39	23.89±1.00	24.81±1.40	24.68±1.47	0.053
Corneal curvature, mm	7.74±0.24	7.73±0.23	7.74±0.25	7.74±0.24	0.989

Values are described as mean ± standard deviation. Analysis of variance (ANOVA) with Bonferroni-adjusted post hoc comparisons was used to investigate the differences of biographical and clinical factors if the P value is more than 0.05 in the test of homogeneity of variances. If not, Tamhane’s T2-adjusted post hoc test was applied.

**Fig 1:**
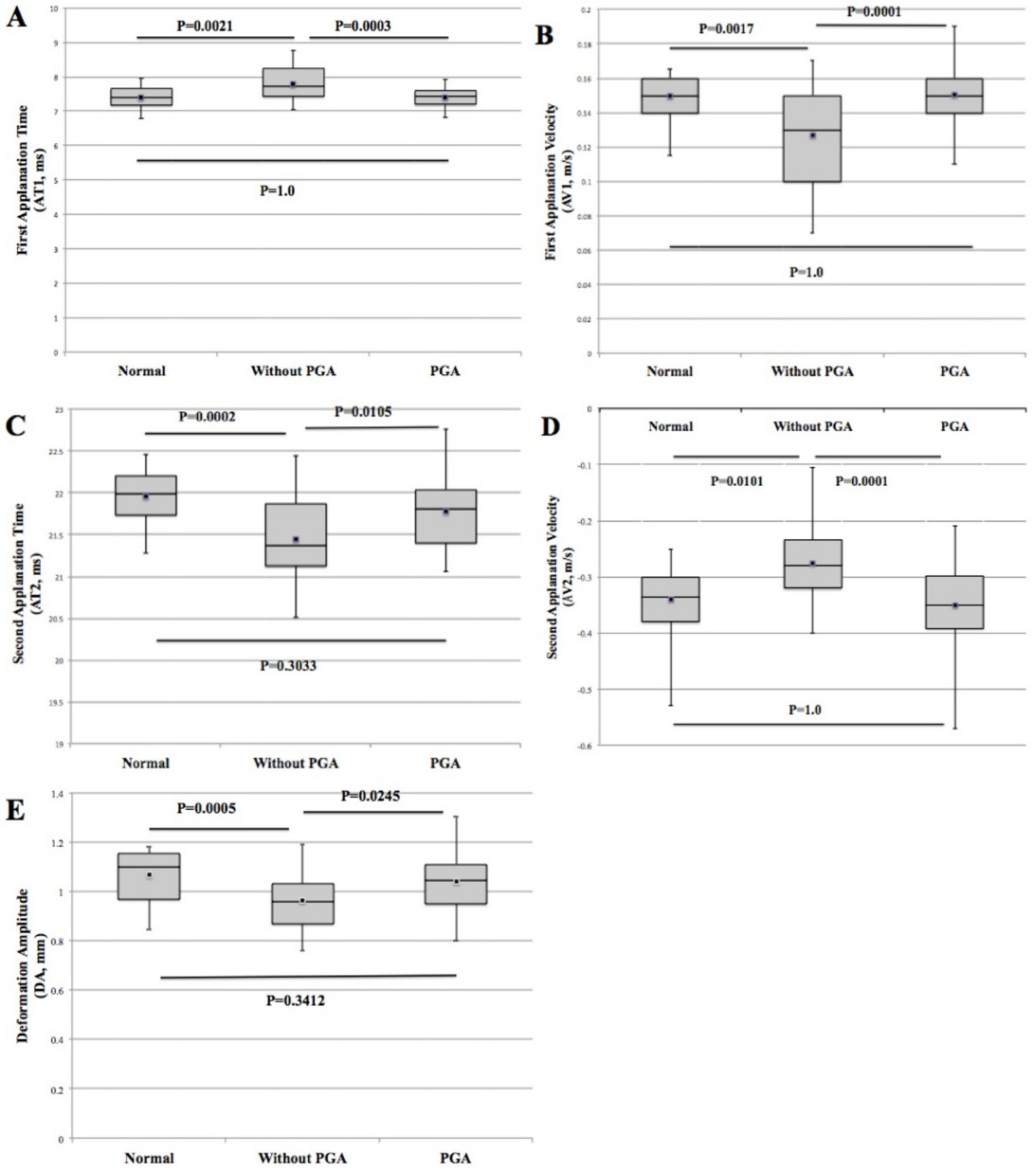
Box-and-whisker plot of the mean AT1 (A), AV1 (B), AT2 (C), AV2 (D) and DA (E) in the normal, glaucoma without PGA treatment and glaucoma with long-term PGA treatment groups. Black squares and thin error bars represent means and maximum/minimum values, respectively. General linear model analysis was performed to investigate the corneal biomechanical property changes among the 3 subgroups. Furthermore, pairwise comparisons using Bonferroni correction for least squares means were employed.

After adjusting for age and gender, time from air-puff starting until the first applanation (AT1, in milliseconds) was longer in newly diagnosed glaucoma patients (7.79±0.47) compared to that in normal controls and patients with long-term PGA therapy (7.41±0.29 and 7.41±0.28, respectively. Table 3, Fig 1A). With the longer time needed in AT1, slower AV1 (in meters/second) was present in newly diagnosed patients (0.13±0.03) than that in the other 2 groups (0.15±0.01, 0.15±0.02, respectively. Table 3, Fig 1B). Contrary to changes in AT1 and AV1, shorter time and quicker velocity were recorded at the second applanation, as represented by AT2 and AV2, respectively, in newly diagnosed patients compared with the other 2 groups (Table 3, Fig 1C and 1D). No statistical significant differences appeared in terms of all corneal biomechanical parameters measured by Corvis ST between the normal controls and glaucoma patients with long-term treatment by PGA (Table 3). Reduced mean amplitude of DA was shown in newly diagnosed glaucoma patients (0.96±0.11), which was significantly lower than that in normal controls (1.07±0.10, p = 0.0005) and in patients who accepted long-term PGA therapy (1.04±0.11, p = 0.0245. Table 3, Fig 1E).

**Table 3:** Corneal biomechanical properties of normal controls, newly diagnosed glaucoma patients without PGA treatment and glaucoma patients with topical long-term PGA therapy after adjusting for age and gender.

	Normal subjects N = 19	Eyes without PGA treatment N = 35	Eyes with PGA treatment N = 34	P value
**AT1**	7.41±0.29	7.79±0.47	7.41±0.28	**<0.0001**
AL1	1.82±0.04	1.81±0.04	1.79±0.10	0.5876
**AV1**	0.15±0.01	0.13±0.03	0.15±0.02	**<0.0001**
**AT2**	21.96±0.31	21.45±0.47	21.78±0.44	**0.0001**
AL2	1.77±0.24	1.79±0.20	1.80±0.22	0.9430
**AV2**	-0.34±0.06	-0.28±0.07	-0.35±0.08	**<0.0001**
HC-time	17.36±0.41	17.18±0.41	17.24±0.60	0.1772
HC-PD	4.60±0.68	4.65±0.33	4.78±0.56	0.4461
HC-radius	7.75±0.82	8.47±1.06	7.89±1.12	0.0313
**DA**	1.07±0.10	0.96±0.11	1.04±0.11	**0.0004**

Values are described as mean ± standard deviation. General linear model analysis was performed to investigate the corneal biomechanical property differences among the 3 subgroups.

**Fig 2:**
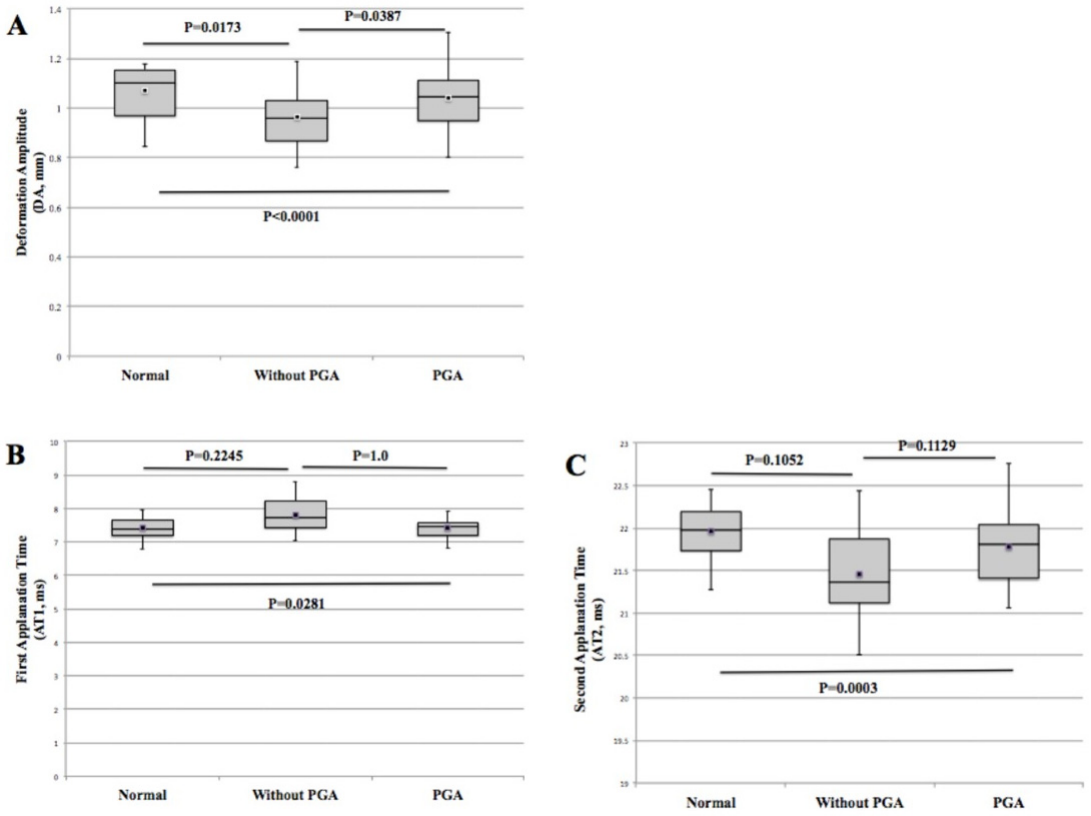
Box-and-whisker plot of the mean DA (A), AT1 (B) and AT2 (C) in the normal, glaucoma without PGA treatment and glaucoma with long-term PGA treatment groups. Black squares and thin error bars represent means and maximum/minimum values, respectively. General linear model analysis was performed to investigate the corneal biomechanical property changes among the 3 subgroups. Furthermore, pairwise comparisons using Bonferroni correction for least squares means were employed.

General linear model analysis indicated that mean DA was the only corneal biomechanical parameter that displayed statistical significant difference among the 3 subgroups (Fig 2A) after adjusting for all biographical and clinical factors, including age, gender, axial length, corneal curvature, IOP, and CCT. The biggest magnitude of DA was achieved in normal eyes, followed by patients with long-term PGA therapy, and then in newly diagnosed glaucoma patients (Fig 2A). In addition, both mean AT1 and AT2 were significantly different between the normal controls and patients with long-term PGA therapy (p = 0.0281 and p = 0.0003, respectively. Fig 2B and 2C, respectively).

We further analyzed the differences of corneal biomechanical properties among the 3 PGA treatment subgroups, namely bimatoprost, latanoprost and travoprost (Table 4). The results showed that after adjusting for age and gender, all corneal biomechanical parameters displayed no statistical significant differences. Similar results were obtained after adjusting for all of the biographical and clinical factors, including age, gender, axial length, corneal curvature, IOP, and CCT.

**Table 4:** Corneal biomechanical properties of POAG patients with long-term treatment of bimatoprost, latanoprost or travoprost.

	Bimatoprost N = 9	Latanoprost N = 9	Travoprost N = 16	P_1_ value	P_2_ value
AT1	7.46±0.13	7.55±0.29	7.31±0.30	0.1316	0.3959
AL1	1.81±0.04	1.83±0.02	1.77±0.14	0.4951	0.8645
AV1	0.15±0.01	0.14±0.02	0.16±0.02	0.0332	0.2802
AT2	21.57±0.31	21.64±0.42	21.97±0.45	0.0815	0.1464
AL2	1.86±0.19	1.72±0.26	1.82±0.21	0.3120	0.2178
AV2	-0.35±0.10	-0.33±0.09	-0.36±0.06	0.3767	0.6711
HC-time	17.46±0.36	16.92±0.55	17.29±0.68	0.3528	0.3006
HC-PD	4.60±0.89	4.63±0.43	4.96±0.30	0.2925	0.2407
HC-radius	8.12±1.11	8.21±1.53	7.58±1.07	0.4963	0.7317
DA	1.03±0.07	0.99±0.11	1.08±0.11	0.2176	0.6385

Values are described as mean ± standard deviation. P_1_ and P_2_ values represented analysis results of general linear model either after adjusting for age and gender, or adjusting for age, gender, axial length, corneal curvature, IOP, and CCT, respectively.

## Discussion

To the best of our knowledge, this is the first study investigating the corneal biomechanical property changes measured by a novel noncontact tonometer (Corvis ST) after long-term treatment by PGA hypotensive medications in glaucoma patients. Our results showed that after adjusting for factors that potentially influence corneal dynamic parameters, significant difference of DA still exists between eyes with at least 2 years of PGA usage and normal eyes as well as newly diagnosed open angle glaucoma eyes. This suggests that PGA may have an affect on corneal biomechanical properties.

Eyes with newly diagnosed open angle glaucoma tended to have significantly less deformable cornea than the other 2 groups. After adjusting for age and gender, it can be seen from our study that AT1 and AV2 were significantly higher, while AT2 and AV1 were significantly lower in the medication naïve group than that in normal subjects and in patients with long-term PGA treatment. Considering that the 3 groups were matched for age, gender and had comparable mean values of CCT, axial length and corneal curvature, the significantly higher IOP values found in the newly diagnosed patients might be the reason why they had less deformable corneas. Smedowski et al reported that a strong correlation exists between the cornea’s dynamic properties, mostly the time and velocity of each cornea deformation and the Corvis ST IOP values, even after standard CCT correction [13]. Huseynova et al discovered that IOPcc was positively correlated with AT1 and AV2, and negatively correlated with AT2 and AV1, and suggested that higher IOP induces a stronger pressure threshold to make the cornea move, and hence a longer time to build that pressure prior to initiation of deformation which gives rise to slower initial velocity [5]. Besides the applanation time and velocity, the DA of the newly diagnosed glaucoma eyes was significantly lower than that in the other 2 groups. It has been reported that IOP is the strongest predictor of DA, followed by other corneal parameters [5]. This indicates that higher values of IOP correlate with smaller magnitude of DA.

Precluding the impact of IOP on corneal biomechanical properties, lots of studies suggested that many other clinical and demographic factors have an effect on corneal dynamics. Higher CCT has been reported to induce a stiffer appearance of the cornea, accompanied with less deformation during the air-pulse [14]. Beyond that, axial length as a more direct variable related to tissue extension and reflection of ocular rigidity [15], was significantly associated with corneal biomechanical properties. Several studies have confirmed that axial elongation was significantly associated with lower CH [16–18]. In addition, CH was significantly associated with corneal curvature, which means flatter corneas are associated with lower CH readings [16, 19]. Furthermore, Narayanaswamy et al. reported that CH was negatively associated with age, corneal radius of curvature and axial length, while positively associated with female gender and CCT [16]. These results indicated that corneal biomechanical properties of hysteresis are significantly influenced by demographic and ocular factors, such as age, gender, IOP, CCT, corneal radius of curvature and axial length.

In view of the potential impacts of the aforementioned ocular factors on corneal biomechanical behaviors, we adjusted for all of the demographic and ocular features to determine whether significant differences of corneal biomechanical properties existed among the 3 groups. Our results revealed that DA showed significant differences between each 2 groups within the total groups. DA has been reported as the best repeatable and reproducible indicator and should be considered as the most valuable for describing corneal biomechanics [11, 20, 21]. In our study, DA in newly diagnosed glaucoma patients was significantly lower than that in the normal eyes, suggesting that diagnosis of POAG perhaps influences corneal biomechanics. This is in agreement with the result obtained from the study of Salvetat et al [11]. They discovered that diagnosis of POAG was significantly correlated with less deformable corneas after excluding the influences from the other variables [11]. It can be found from our study that eyes with long-term PGA treatment had significantly higher DA than in treatment naïve patients, suggesting that long-term treatment by PGA perhaps directly increases corneal deformation property.

In order to investigate if there are any inconsistences caused by different types of PGA, we further analyzed the corneal biomechanical property changes based on the type of PGA. Limited to the small number of each subgroup, we found that there were no statistical significant differences in all of the corneal biomechanical parameters among the 3 subgroups. This is also the first study to compare the corneal biomechanics changes under different PGA medications. Large scale and prospective study are needed to investigate if there are any corneal biomechanical property changes caused by different PGA medications.

Investigations concerning the effects of PGA on corneal biomechanical properties are rare and have all used ORA as a survey tool to measure corneal biomechanical changes. It has been reported that baseline CH was a significant predictor of the amplitude of IOP decrease [12, 22]. The prospective study of Tsikripis et al. showed that IOP reduction under 3 years topical PGA therapy was accompanied with increased CH [9]. Bolivar et al. discovered that treatment with latanoprost increases CH, which reflects corneal viscoelastic properties and provides information on corneal biomechanical behavior [6]. This increase was not related to IOP decrease, suggesting a direct effect of PGA on the viscoelastic corneal properties [6], which was in accordance with our study.

It has been generally acknowledged that long-term topical PGA therapy has an influence on CCT. Several studies have reported that the mean CCT was significantly reduced in the first 2 years of treatment [3, 23]. Hence, 2 years was chosen as a time point in our study. Our results revealed that even though there was a slightly decrease in CCT after at least 2 years of PGA treatment compared to normal controls and newly diagnosed patients, the difference was not statistically significant. It was theorized that PGA acts primarily through activation of matrix metalloproteinases(MMPs) with resultant degradation of the cornea stromal extracellular matrix, which ultimately leads to CCT reduction [3, 23]. Furthermore, PGA may increase the keratocyte density in the corneal stroma, perhaps due to the activation of MMPs and suppression of tissue inhibitors of metalloproteinases [22]. Hence, a plausible explanation for the PGA-induced direct influence on DA may be because of the effects of the drugs on cornea tissue remodelling. However, the exact mechanisms of the effects on DA induced by PGA still needed to be studied in the future.

The small sample size is a limitation of this study and the different types of PGA within the medication treatment group could possibly be an unknown variance. In addition, in view of the cross-sectional nature of this investigation, prospective studies will be needed to further confirm the conclusions.

In conclusion, an important finding in our study is that PGA seems to have an effect on corneal biomechanical properties in addition to the indirect effect owing to the PGA-induced IOP reduction and CCT decrease on the corneal dynamic properties. Our results can contribute to a broader understanding of the effects of PGA on corneal biomechanical properties. Also, long-term usage of topical PGA therapy needs to be taken into account when measuring the corneal biomechanical properties in glaucoma patients.
